# Supplementary material for: Structural conversion of the spidroin C-terminal domain during assembly of spider silk fibers
Source: Nat Commun. 2024 May 31;15:4670. doi: 10.1038/s41467-024-49111-5 (PMC11143275; doi:10.1038/s41467-024-49111-5)
Supplement: Supplementary file 1 — Supplementary information [file 41467_2024_49111_MOESM1_ESM.pdf]

Supporting Information

Untangling spider silk secrets: The structural basis of alpha-helix to beta-sheet conversion of the spidroin C-terminal domain during fiber assembly

Danilo Hirabae De Oliveira (1,2), Vasantha Gowda (2), Tobias Sparrman (3), Linnea Gustafsson (4), Rodrigo Sanches Pires (2), Christian Riekel (5), Andreas Barth (6), Christofer Lendel (2)*, My Hedhammar (1)*

*Corresponding Authors: My Hedhammar – Email: myh@kth.se; Christofer Lendel: lendel@kth.se

1. Department of Protein Science, School of Engineering Sciences in Chemistry, Biotechnology and Health, KTH Royal Institute of Technology, AlbaNova University Center, Stockholm, Sweden, 2. Department of Chemistry, KTH Royal Institute of Technology, Stockholm, Sweden, 3. Department of Chemistry, Umeå University, Umeå, Sweden. 4. Spiber Technologies AB, Roslagstullsbacken 15, Stockholm, 114 21 Sweden, 5. European Synchrotron Radiation Facility, B.P. 220, F-38043 Grenoble Cedex France. 6. Department of Biochemistry and Biophysics, Stockholm University, Stockholm, Sweden.

*Corresponding Authors: My Hedhammar – Email: myh@kth.se; Christofer Lendel: lendel@kth.se

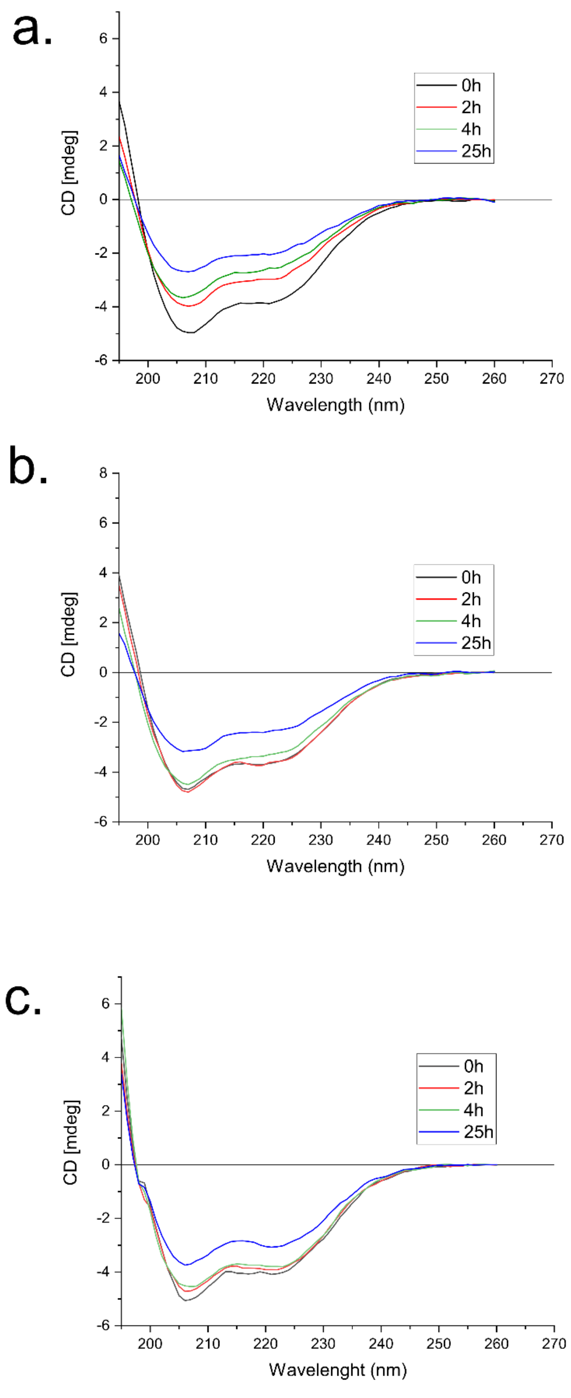

Figure S1. Circular dichroism spectra of the CT domain in solution. CD spectra of the CT domain at three different pH conditions: a) pH 4, b) pH 8 and c) pH 12, all display α-helical patterns. The α-helical proteins commonly show negative bands at 222 nm and 208 nm minima and a positive band at 193 nm)[1].

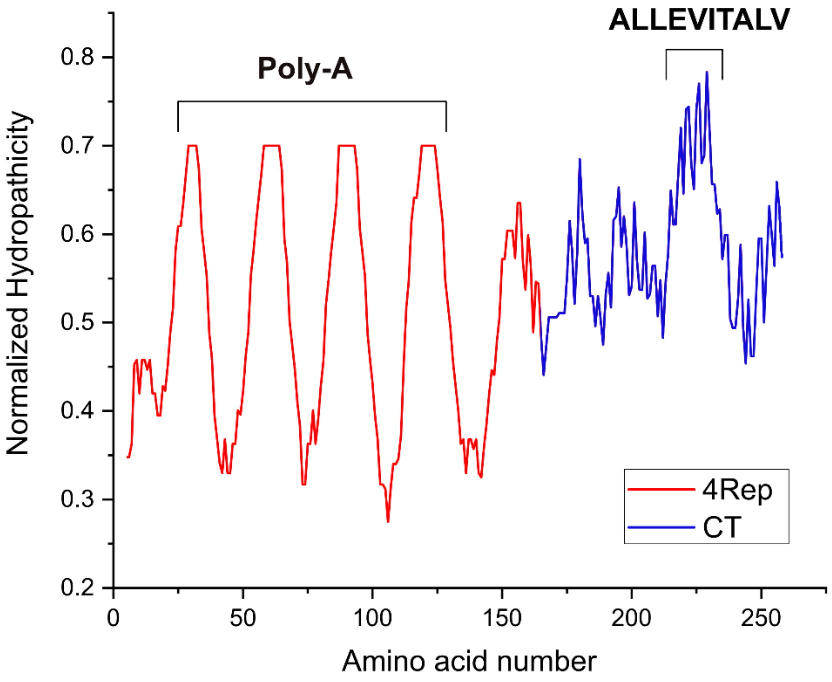

10 20 30 40 50 60
GPNSGQGGYG GLGQGGYGQG AGSSAAAAAA AAAAAAGQGG QGQGGYGQGS GGSAAAAAAA

70 80 90 100 110 120
AAAAAAAAGR GQGGYGQGSG GNAAAAAAAA AAAAAAAGQG GQGGYGRQSQ GAGSAAAAAA

130 140 150 160 170 180
AAAAAAAAGS GQGGYGGQGQ GGYGQSSASA SAAASAASTV ANSVSRLSSP SAVSRVSSAV

190 200 210 220 230 240
SSLVSNGQVN MAALPNIISN ISSSVSASAP GASGCEVIVQ ALLEVITALV QIVSSSSVGY

250 260
INPSAVNQIT NVVANAMAQV MG

Figure S2. Normalized Kyte & Doolittle hydropathicity of 4RepCT sequence. The 4Rep segment is presented in red and the CT domain is presented in blue. For the synchontron X-ray diffraction experiment, the CT construct had a slightly extended sequence (underlined red segment).

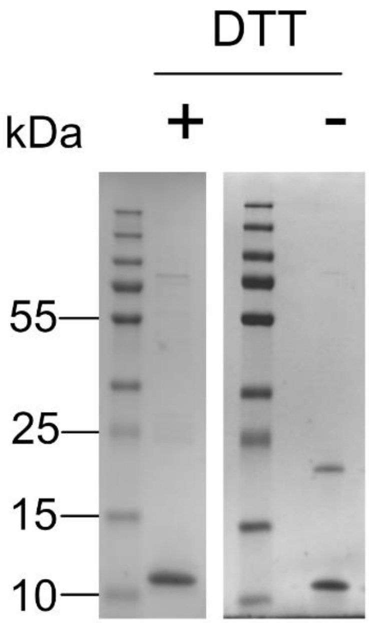

Figure S3. SDS-PAGE analysis of the denatured soluble CT domain with (+) and without (-) the reducing agent dithiothreitol (DTT).

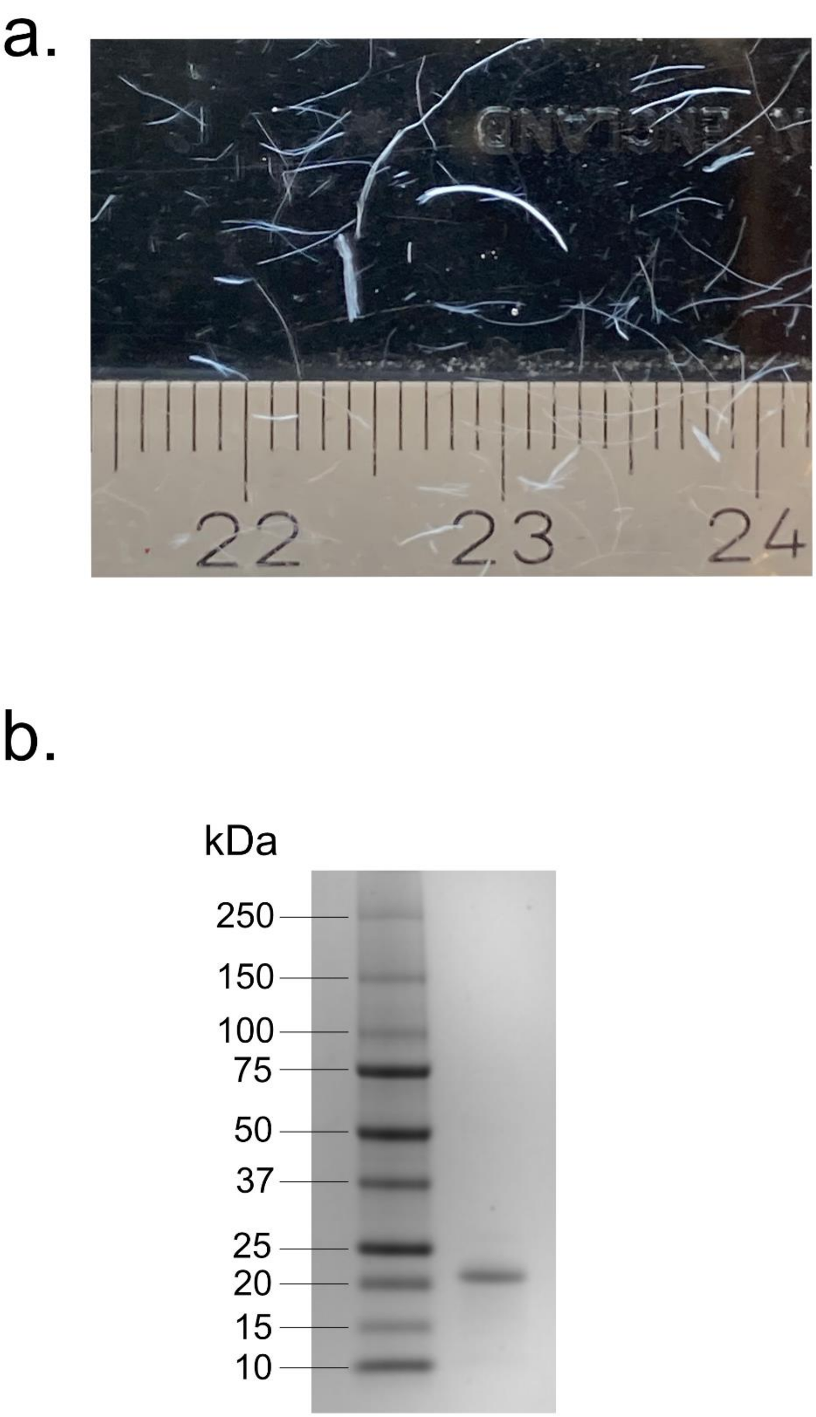

Figure S4. SDS-PAGE analysis of the denatured CT domain fibers. The CT domain fibers shown in (a) were collected and dissolved in 6 M urea before SDS-PAGE analysis (b) without reducing agent. The obtained band corresponds to the approximate molecular size of 20 kDa, i.e. a dimer.

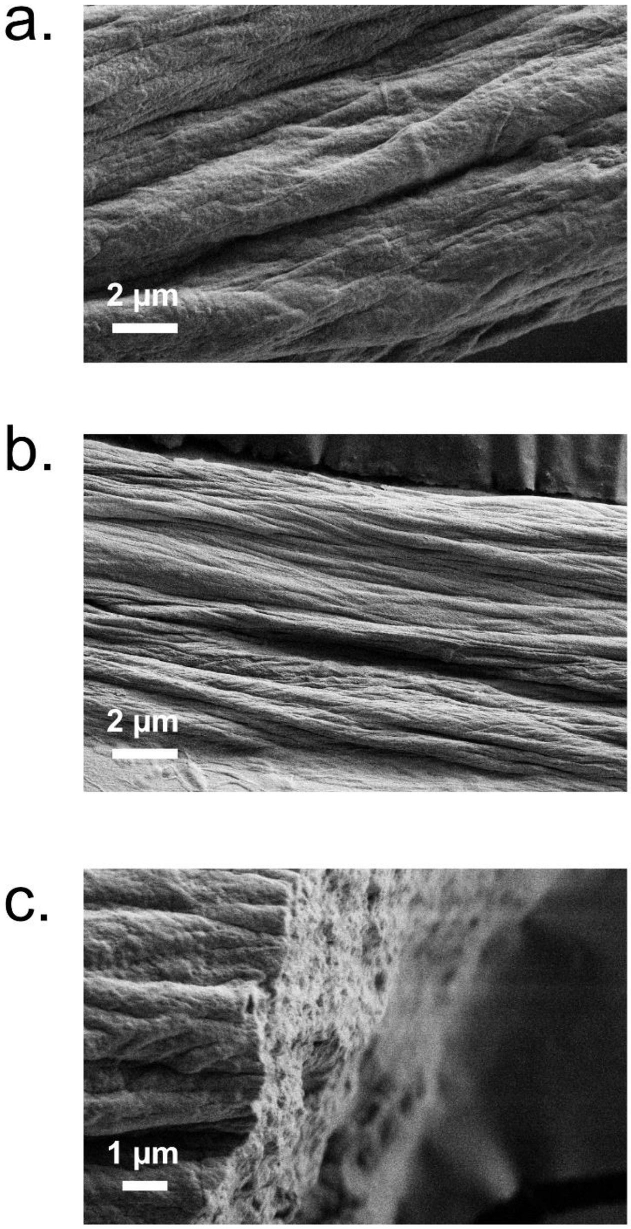

Figure S5. Scanning electron microscopy of a CT domain fiber bundle. a) and b) Surface analysis of the CT fibers show oriented striated-like silk morphology and length similar to that of 4RepCT fibers from previous studies[2]. c) The micrograph of the interior core of a fractured CT fiber. The fibrillar structures are comparable with previous SEM analysis of recombinant silk[3] and natural silk[4].

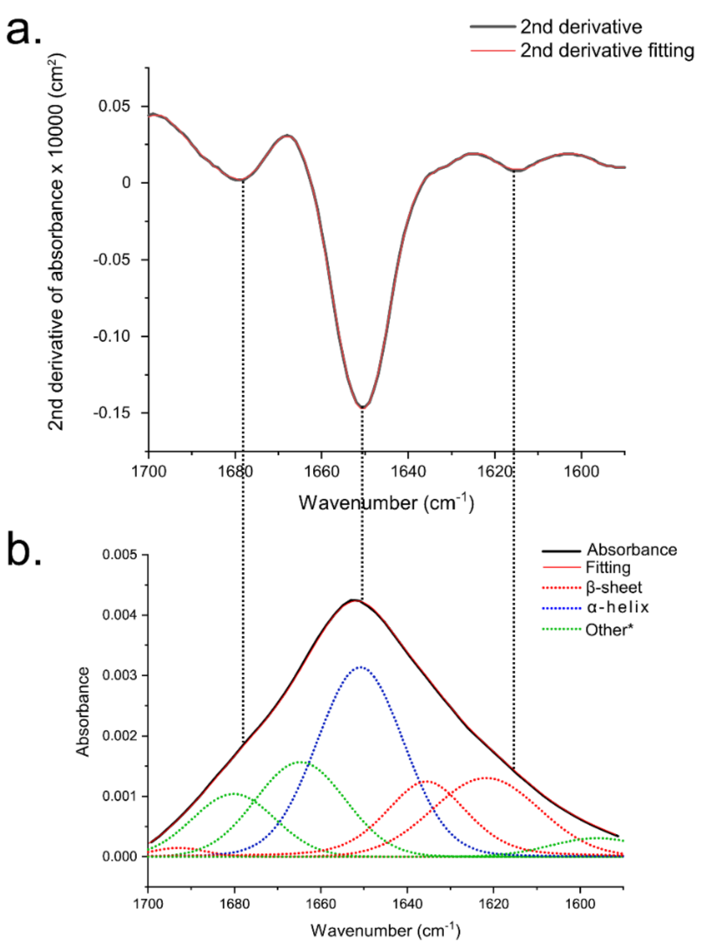

Figure S6. Infrared spectroscopy of CT in the amide I region. The top panel (a) presents the second derivative of the absorbance spectrum (black) and the respective fit (red) for the dried drop of CT. The bottom panel (b) presents the absorbance spectra and the fitted component bands. The IR spectra corroborates the secondary structure change upon transition from soluble CT domain to solid CT silk fiber. *All other secondary structures (irregular, turns, bends and other helix types).

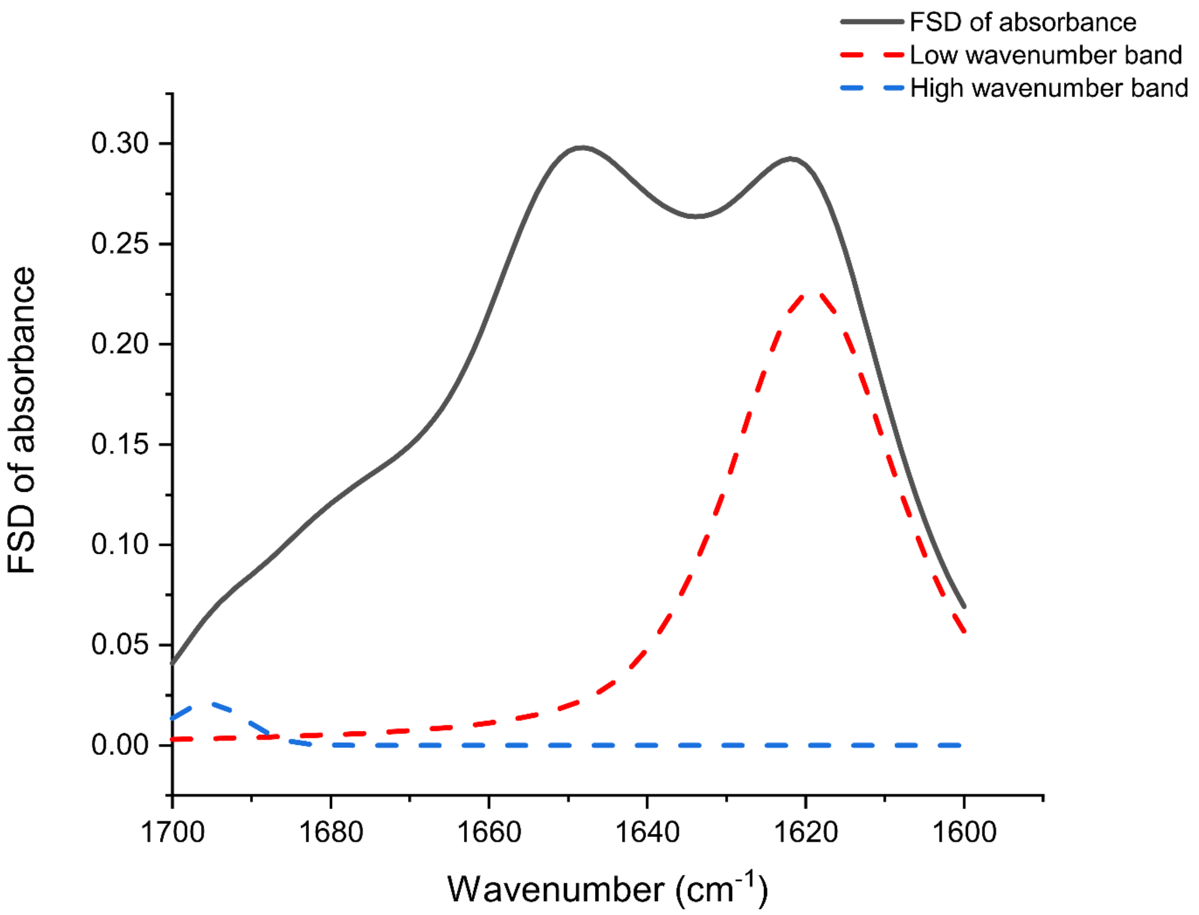

Figure S7. Fourier self-deconvolution (FSD) of the absorbance spectrum of the CT fiber and the fitted component bands assigned to β-sheets. The deconvolution and the fitting were done to calculate the β-sheets organizational index.

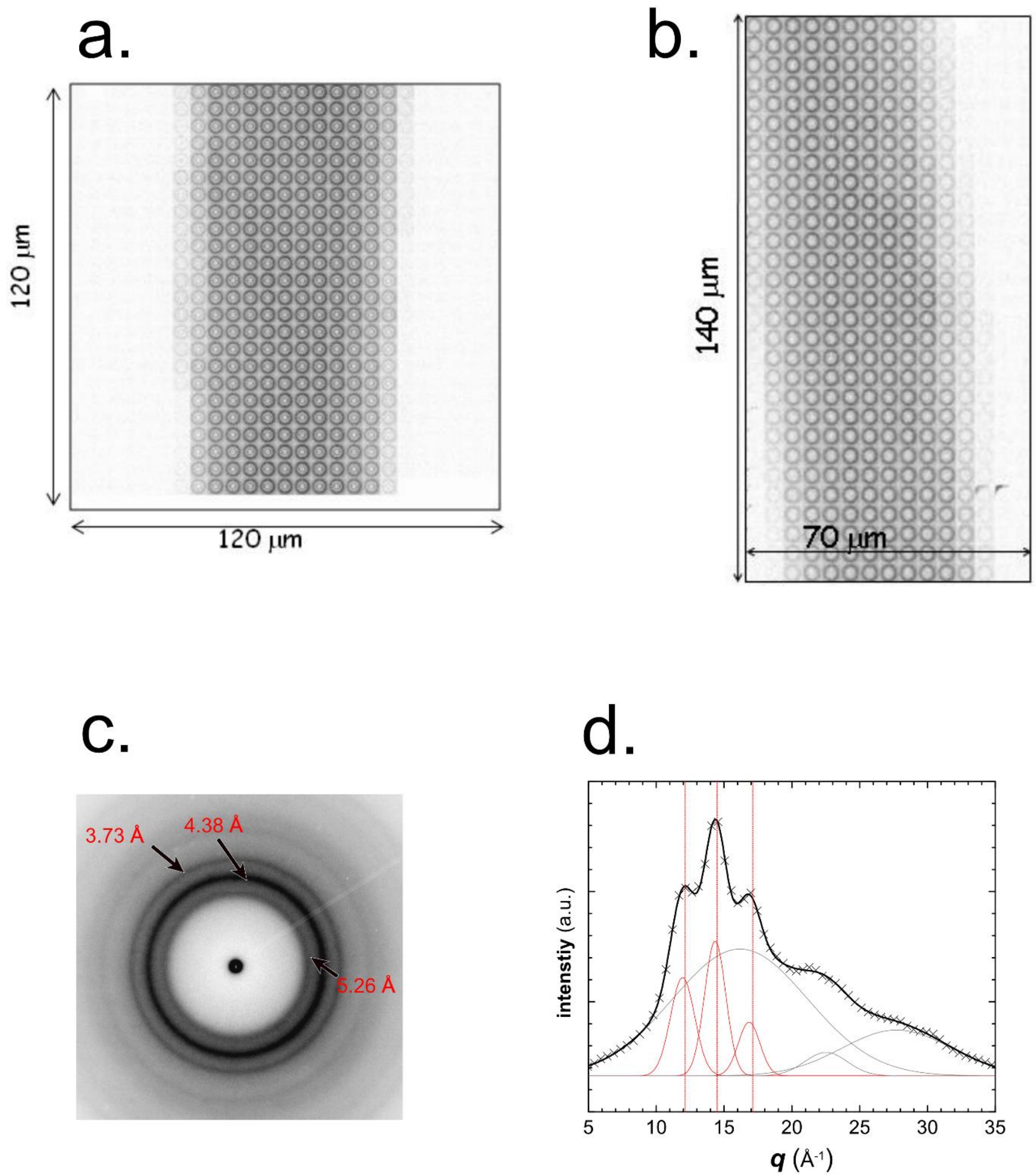

Figure S8. Additional x-ray diffraction analyses. Raster-scans of CT domain (a) and 4RepCT (b) silk fibers. Composite image of CCD-patterns ('pixels') obtained from the raster-scans. Note that the scale of all composite images is the same. The X-ray diffraction pattern of aggregated 4Rep: individual diffraction pattern (c) and azimuthally averaged pattern (c).

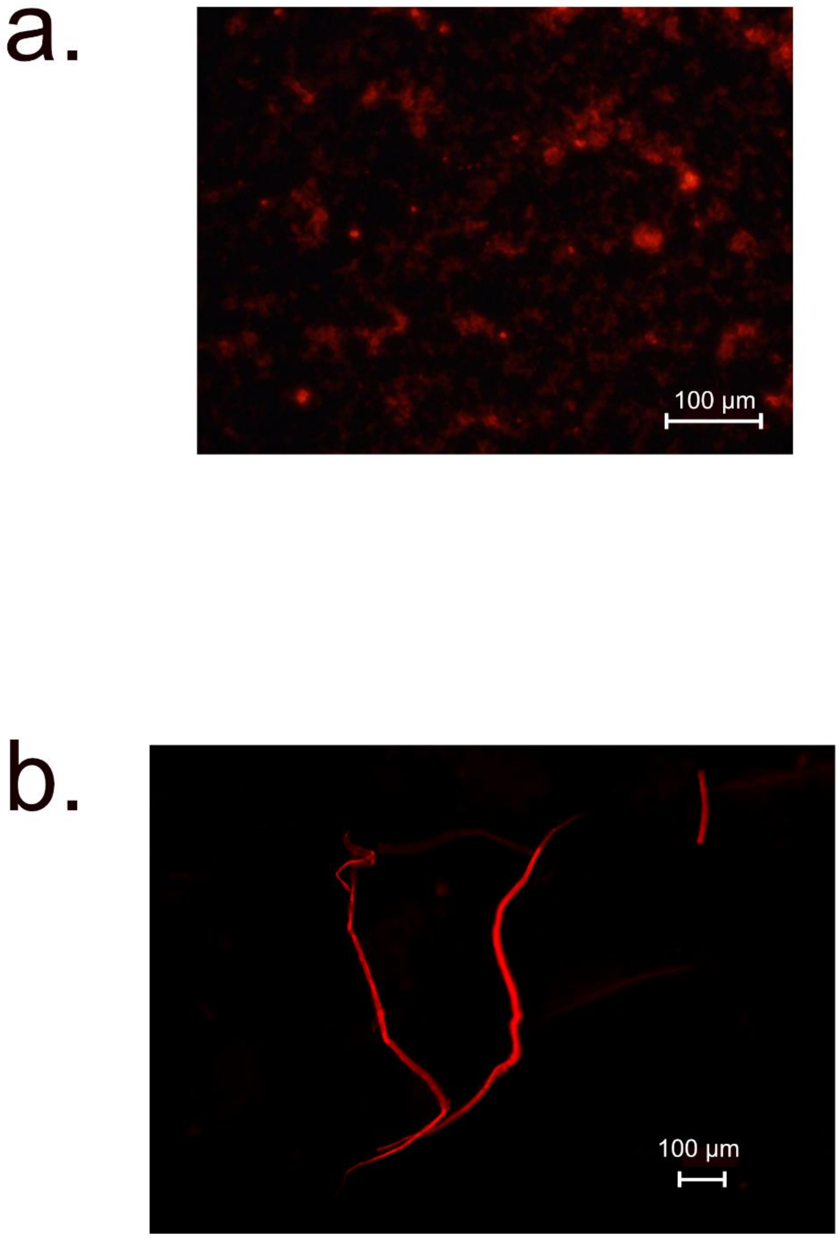

Figure S9. Fluorescence microscopy and β-sheet structure detection: a) Peptide of helix Nº4 in solution with the Amytracker 630 dye. b) A CT fiber with the Amytracker 680 dye.

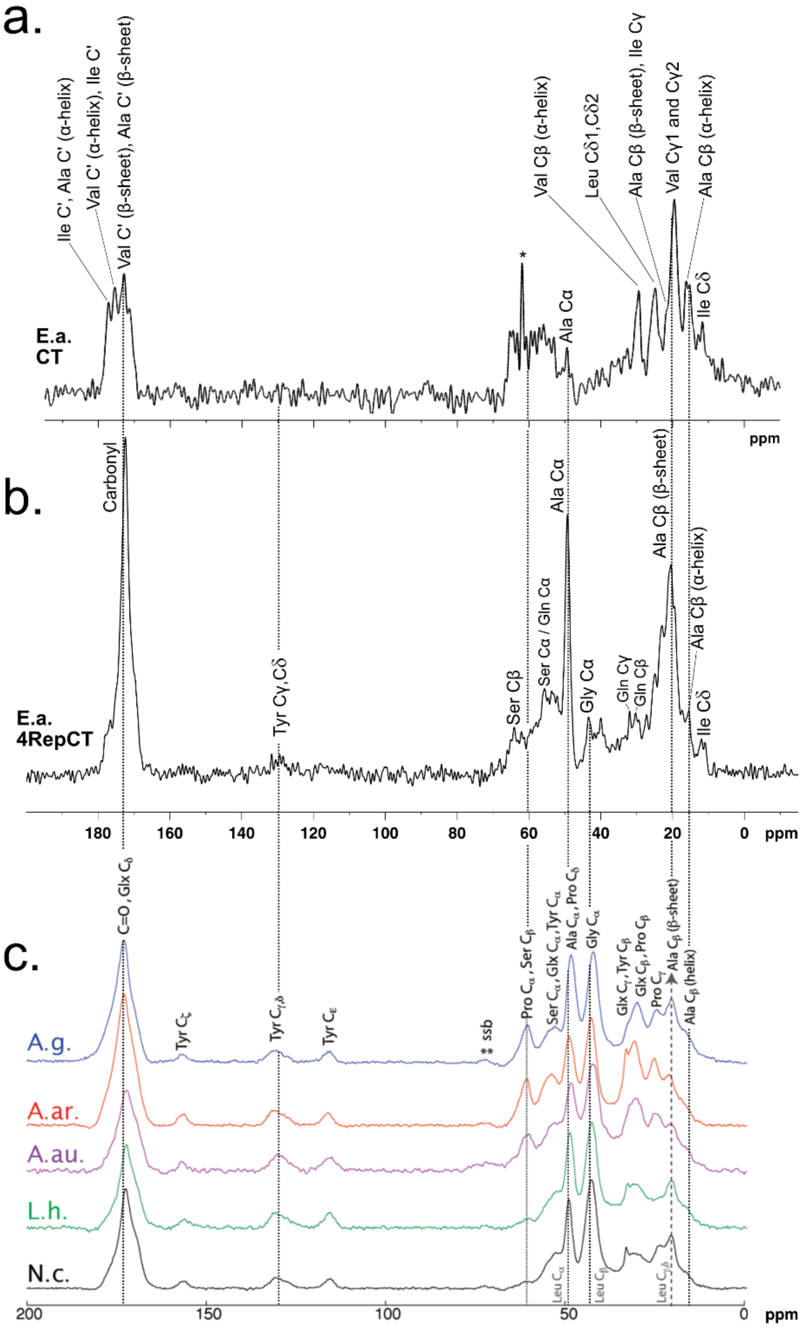

Figure S10. Natural abundance ^{13}C CP-MAS spectra of different silk fibers. a) Recombinant CT domain and b) 4RepCT from *Euprosthenops australis* (E.a.), in fiber form. c) *Araneus gemmoides* (A.g.), *Argiope argentata* (A.ar), *Argiope aurantia* (A.au), *Latrodectus Hesperus* (L.h.), and *Nephila clavipes* (N.c.) Creager *et al.***.** Reprinted with permission from Creager, M. S., Jenkins, J. E., Thagard-Yeaman, L. A., Brooks, A. E., Jones, J. A., Lewis, R. V., Holland, G. P., & Yarger, J. L. (2010). Solid-state NMR comparison of various spiders' dragline silk fiber. Biomacromolecules, 11(8). https://doi.org/10.1021/bm100399x. Copyright 2021 American Chemical Society.

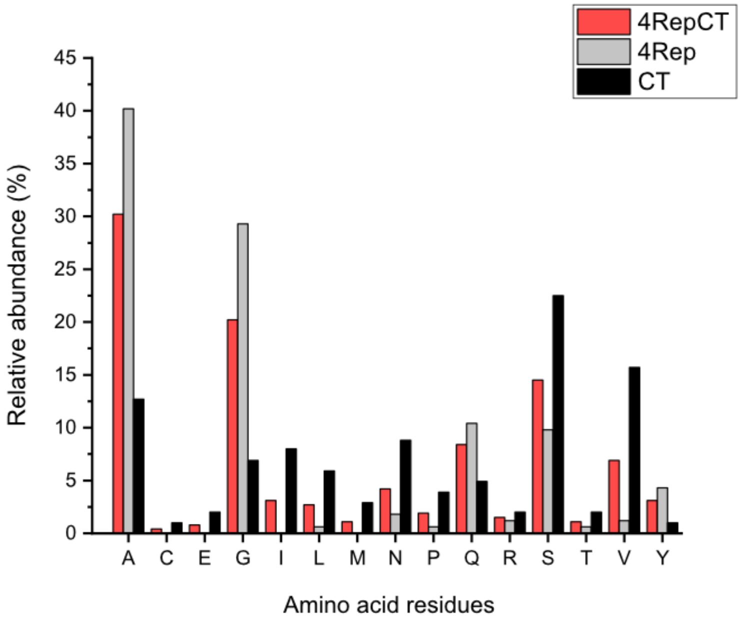

Figure S11. Relative abundance of the different amino acid residues in the recombinant silk constructs: 4RepCT (Red), 4Rep (Grey) and CT domain (Black).

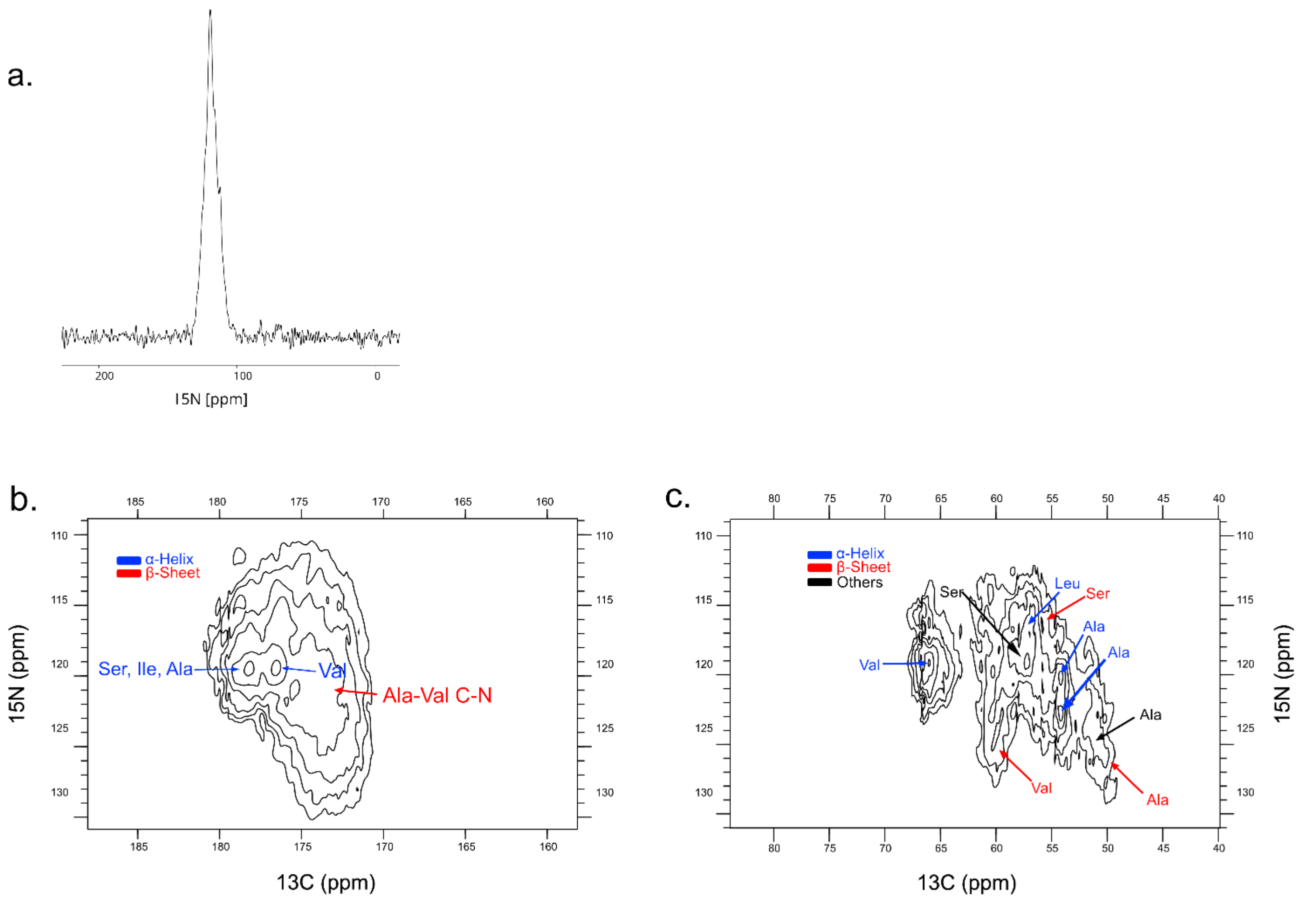

Figure S12. The ^{15}N ssNMR spectrum of the CT fibers has poorly dispersed peaks. a) 1D ^{15}N CP-MAS spectrum. b) 2D HNCO spectrum. c) 2D HNCA spectrum. Assignments of some amino acid types with corresponding secondary structures are indicated.

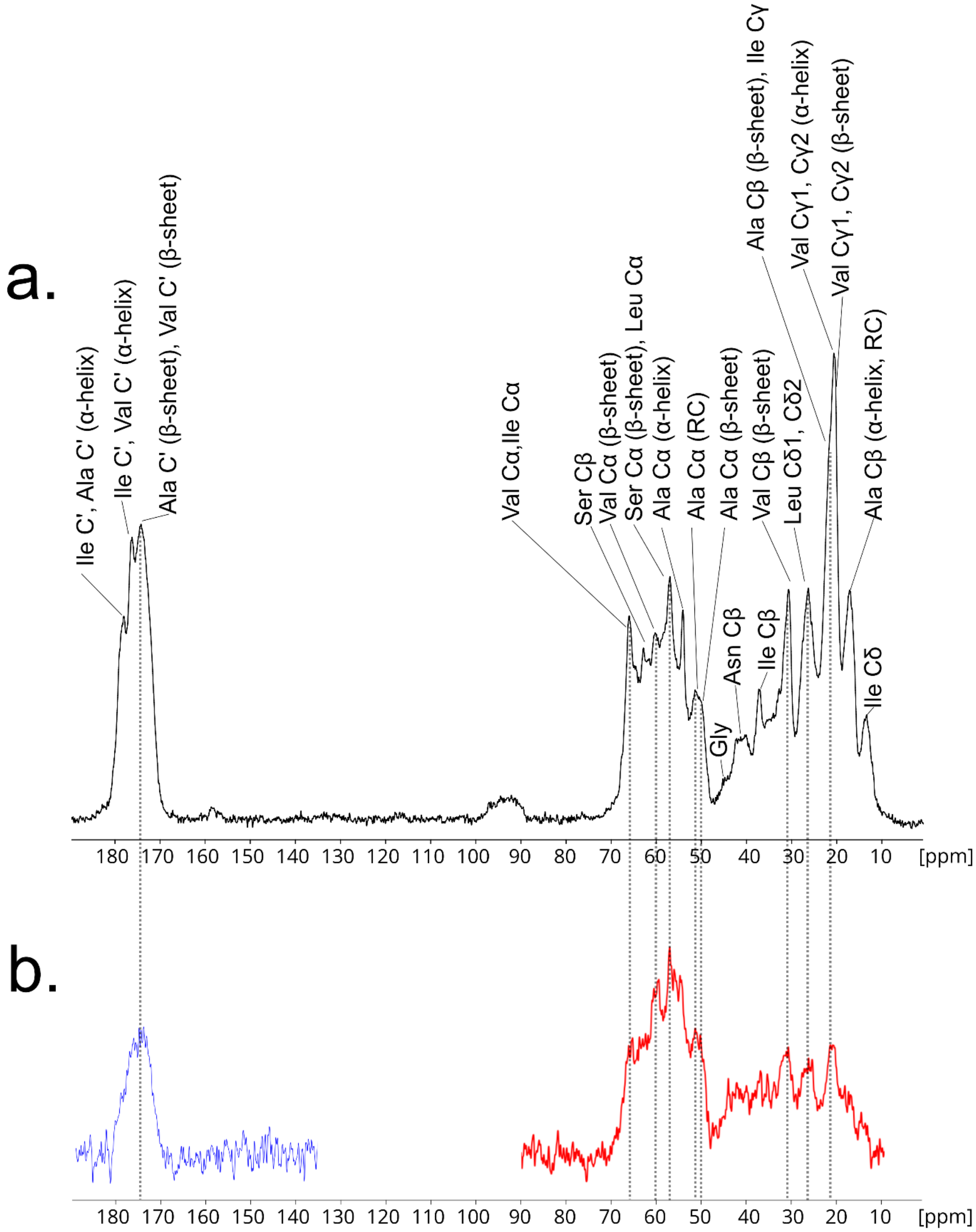

Figure S13. Intermolecular contacts from ssNMR. a) ^{13}C CP-MAS spectrum of $^{13}C^{15}N$-labelled CT. b) 1D PAIN-CP spectra for carbonyl (blue) and aliphatic regions (red), respectively. The peaks assigned to have intermolecular contacts according to the 1D PAIN experiment are indicated with dashed lines.

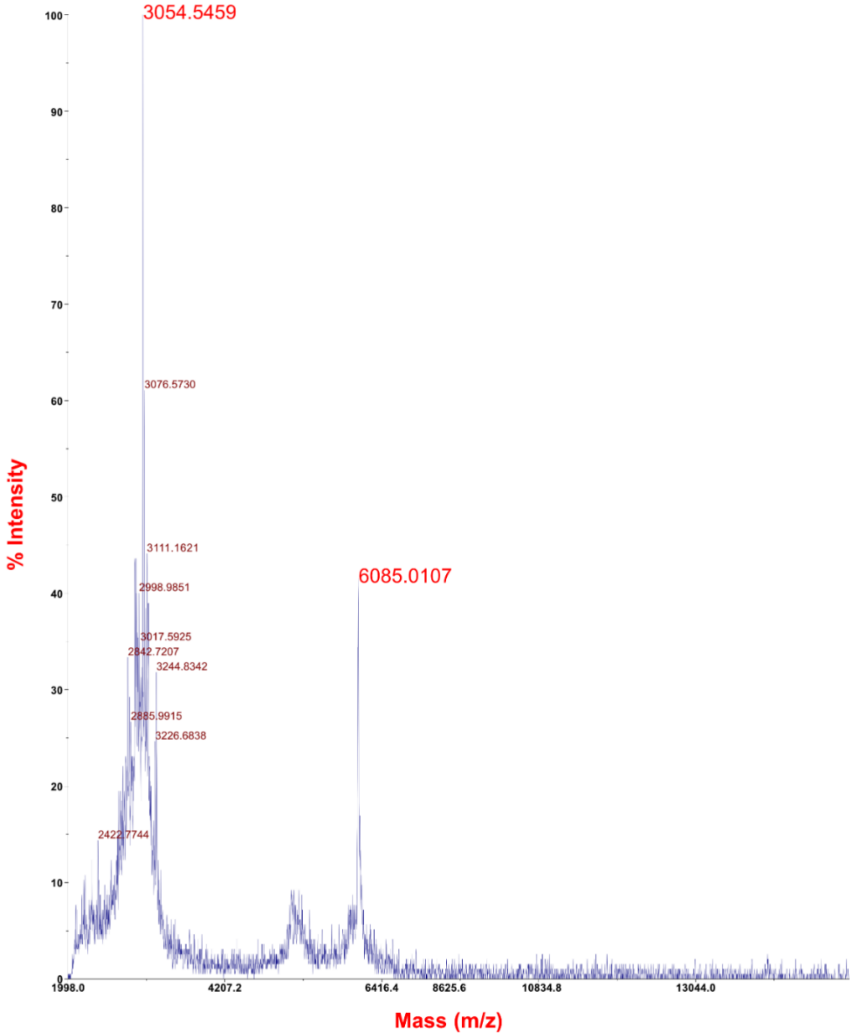

Figure S14. Analysis of the helix Nº4 peptide in pH 2 using MADI-TOF mass spectrometry.

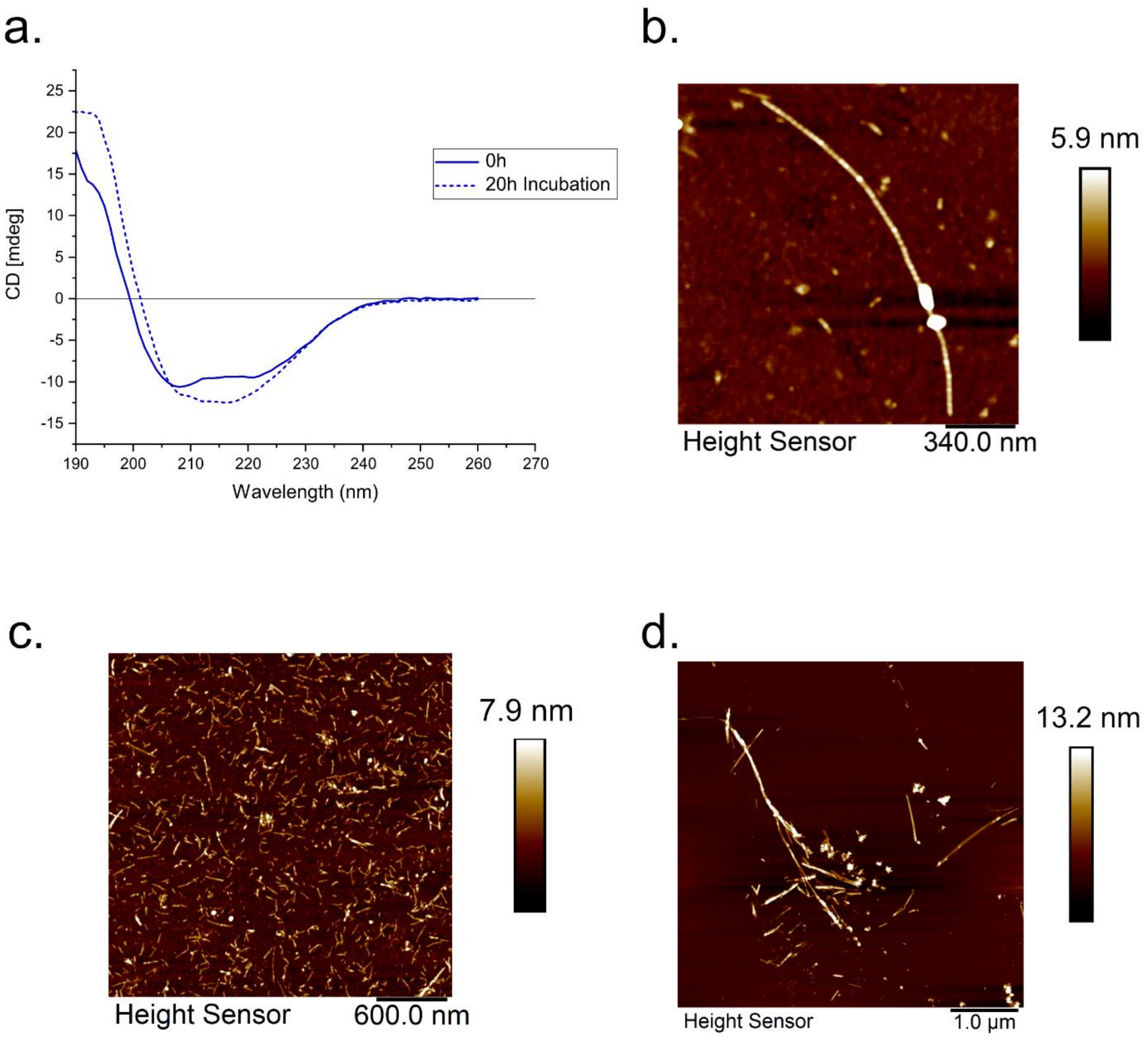

Figure S15. The secondary structure evaluation of helix Nº4 of the CT domain suggests a structural shift to β-sheet. a) CD spectra of the CT_{51-80} peptide at pH 8.1 immediately measured post-solubilization and after incubation at room temperature for 20 h. b) and c) The CT_{51-80} peptide forms nanofibrils at pH 3.1, confirmed by AFM. d) The peptide forms similar at fibrils at pH 2.

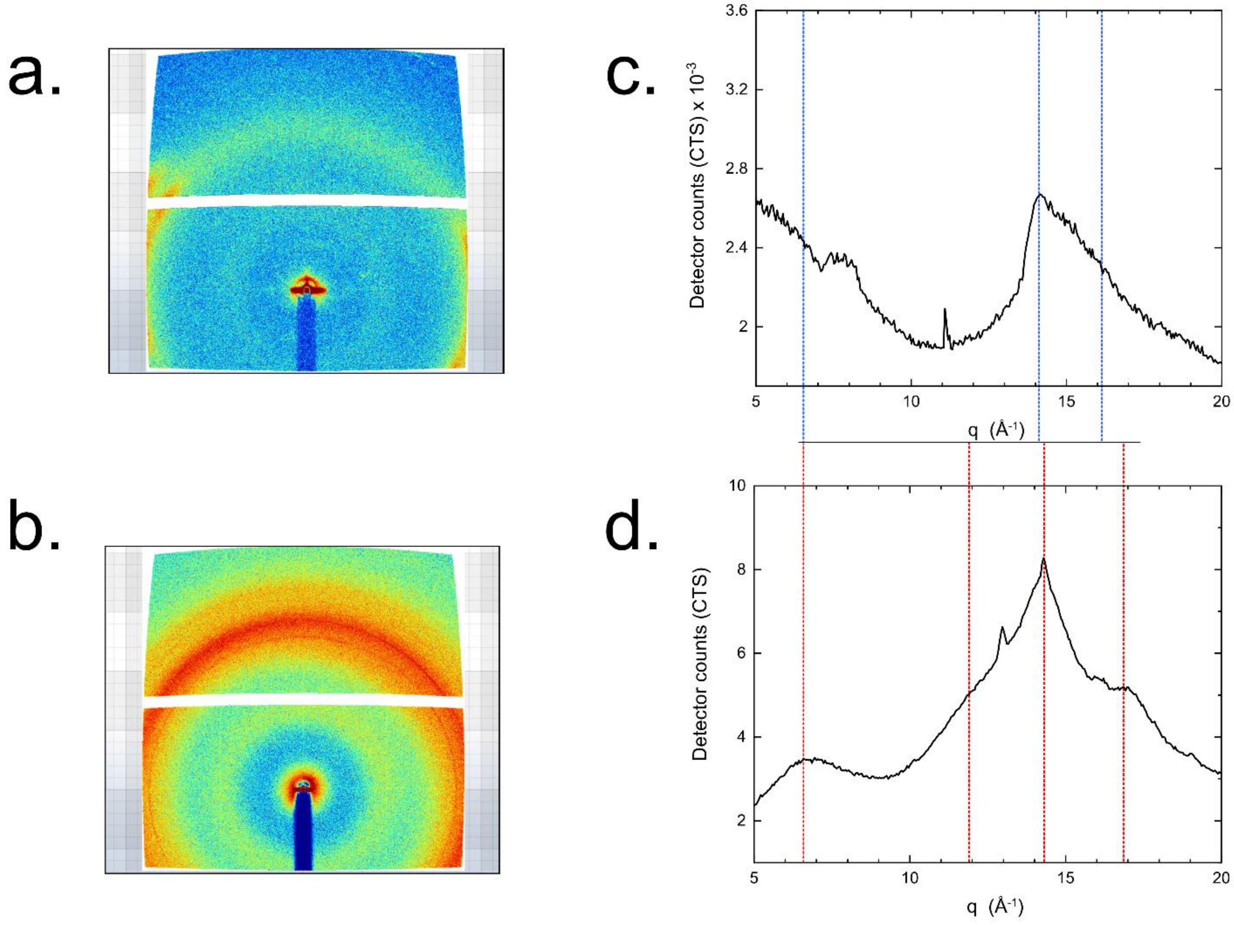

Figure S16. Complementary X-ray diffraction experiments on silk fibers. Individual diffraction patterns (left) and azimuthally averaged pattern (right) of fibers from the CT domain (a. and c.) and the 4RepCT construct (b. and d.). The diffraction patterns are in agreement with those from the synchrotron experiments.

Table S1. CD spectra analysis, deconvolution analysis of CT domain in solution at pH 8.0 using the BeStSel[5] software. *All other secondary structures (irregular, bends, other helix types).

Protein Secondary structure	Content (%)
α-helix	60.8%
β-sheet	13.8%
Turns	3.1%
Other*	22.3%

Table S2. Amide 1 positions assigned to secondary structure elements and relative area found from curve fitting of the spectra of CT domain in fiber form (a) and dried drop (b).

a.

CT Silk fiber					
**Center (cm^{-1})**	**Secondary structure assignment**	**Absorbance**	**FWHH* (cm^{-1})**	**fg**	**Relative Area (%)**
1696	β-sheet	0.0138	15.0	0.54	1.9
1687	Other	0.0292	18.7	1.00	4.2
1673	Other	0.0625	23.2	1.00	11.3
1650	α-helix	0.1717	29.7	1.00	39.6
1630	β-sheet	0.0907	23.0	1.00	16.2
1618	β-sheet	0.0556	18.9	1.00	8.1
1611	β-sheet	0.0779	30.4	1.00	18.4

b.

CT domain Silk dried drop					
**Center (cm^{-1})**	**Secondary structure assignment**	**Absorbance**	**FWHH* (cm^{-1})**	**fg**	**Relative Area (%)**
1692	β-sheet	0.0001	14.5	1.00	1.0
1680	Other	0.0010	22.4	1.00	10.7
1664	Other	0.0016	24.3	1.00	17.6
1650	α-helix	0.0031	23.6	0.93	35.2
1635	β-sheet	0.0012	21.9	0.66	14.5
1621	β-sheet	0.0013	29.1	1.00	17.4
1598	Other	0.0003	25.4	1.00	3.6

* FWHH, full width at half height. The parameter fg indicates the fractional contribution of the Gaussian line shape to the overall line shape. 1-fg is the contribution of the Lorentzian line shape function. *Other* means all other secondary structures (irregular, turns, bends, other helix types).

Table S3. Summary of observed d-spacings from WAXS experiments on fibers from CT domain and 4RepCT, respectively, in comparison to literature data from β-poly(L-alanine).* Presented as 4Rep aggregates.

**Orientation**	**Inter-sheet, Bulky residues**	**Inter-sheet, Poly-A**	**Interchain**	**Intrachain Cα**
Sample	d (Å)	d (Å)	d (Å)	d (Å)
CT	9.75	-	4.59	3.88
4RepCT	9.67	5.17	4.41	3.74
4Rep*	-	5.26	4.38	3.73
β-poly(L-alanine)	-	5.1 (020)	4.3 (210)	3.5 (211)

Table S4. Summary of observed chemical shifts of the CT domain fibers with secondary structure assignments.

Residue type	Recombinant CT fiber protein								
	β-sheet			α-helix			r.c		
	Cα	Cβ	C'	Cα	Cβ	C'	Cα	Cβ	C'
Ser	56.1	64.6	172.2	57.3	62.6	-	-	-	-
Val	59.9	33.7	173.8	65	30.2	177.0	63.7	30.4	-
Ala	50.1	21.5	174.2	54	17.3	177.3	51.2	18.6	177.1
Asn	51.5/51.9	41.6/39.2		55.0	36.8	-	-	-	-
Ile	59.1	40.0		65.6/64.3	46.8/37.1	177.8	-	-	-
Gly	44.2/45.2	-	173.3	-	-	-	-	-	-
Leu	-	-	-	56.9	40.2/40.4	178.2	-	-	-
Gln	53.6	31.5	-	-	-	-	-	-	-
Pro	61.8	31.2	-	-	-	-	-	-	-
Met	-	-	-	57.5/59.2	32.5/35.1	177.8	-	-	-
Glu	53.6	33.1	-	-	-	-	-	-	-
Arg	-	-	-	-	27.3	-	-	-	-
Thr	62.4	69.5	-	68.4	-	-	-	-	-
Cys	-	-	-	-	-	-	-	-	-
Tyr	-	-	-	-	-	-	-	-	-

Table S5. Summary of observed chemical shifts of CT domain fiber and secondary structure correlation with the amino acid position regarding the helical sites (H1 to H5) of the soluble protein.

Amino Acid	Total	α-helix	β-sheet	coil	H1	H2	H3	H4	H5
**Glu**	2		x					2	
**Gln**	5		x				2	2	
**Asn**	9	x	x		1	1	2		1
**Arg**	2	x			1	1			
**Pro**	4	x	x				1		
**Leu**	6	x			1	1	1	3	
**Ile**	8	x	x				3	3	1
**Met**	3	x							2
**Thr**	2	x		x				1	1
**Cys**	1							1	
**Val**	16	x	x	x		4	1	4	4
**Ser**	23	x	x	x	3	7	6	2	
**Ala**	13	x	x	x		2	2	2	2
**Gly**	7		x		1	1		1	
**Tyr**	1							1	

References

1. Holzwarth, G. & Doty, P. The Ultraviolet Circular Dichroism of Polypeptides. *J Am Chem Soc* **87**, 218-28 (1965).
2. Hedhammar, M., Rising, A., Grip, S., Martinez, A.S., Nordling, K., Casals, C., Stark, M. & Johansson, J. Structural properties of recombinant nonrepetitive and repetitive parts of major ampullate spidroin 1 from Euprosthenops australis: implications for fiber formation. *Biochemistry* **47**, 3407-17 (2008).
3. Xia, X.X., Qian, Z.G., Ki, C.S., Park, Y.H., Kaplan, D.L. & Lee, S.Y. Native-sized recombinant spider silk protein produced in metabolically engineered Escherichia coli results in a strong fiber. *Proc Natl Acad Sci U S A* **107**, 14059-63 (2010).
4. Jin, H.J. & Kaplan, D.L. Mechanism of silk processing in insects and spiders. *Nature* **424**, 1057-61 (2003).
5. Micsonai, A., Moussong, E., Wien, F., Boros, E., Vadászi, H., Murvai, N., Lee, Y.H., Molnár, T., Réfrégiers, M., Goto, Y., Tantos, A. & Kardos, J. BeStSel: webserver for secondary structure and fold prediction for protein CD spectroscopy. *Nucleic Acids Res* **50**, W90-W98 (2022).
