# Supplementary material for: Structural conversion of the spidroin C-terminal domain during assembly of spider silk fibers
Source: Nat Commun. 2024 May 31;15:4670. doi: 10.1038/s41467-024-49111-5 (PMC11143275; doi:10.1038/s41467-024-49111-5)
Supplement: Supplementary file 3 — Reporting Summary [file 41467_2024_49111_MOESM3_ESM.pdf]

nature portfolio

Corresponding author(s): My Hedhammar, Christofer Lendel

Last updated by author(s): May 5, 2024

Reporting Summary

Nature Portfolio wishes to improve the reproducibility of the work that we publish. This form provides structure for consistency and transparency in reporting. For further information on Nature Portfolio policies, see our Editorial Policies and the Editorial Policy Checklist.

Statistics

For all statistical analyses, confirm that the following items are present in the figure legend, table legend, main text, or Methods section.

n/a	Confirmed	
☐	☒	The exact sample size (*n*) for each experimental group/condition, given as a discrete number and unit of measurement
☒	☐	A statement on whether measurements were taken from distinct samples or whether the same sample was measured repeatedly
☒	☐	The statistical test(s) used AND whether they are one- or two-sided<br>*Only common tests should be described solely by name; describe more complex techniques in the Methods section.*
☒	☐	A description of all covariates tested
☒	☐	A description of any assumptions or corrections, such as tests of normality and adjustment for multiple comparisons
☒	☐	A full description of the statistical parameters including central tendency (e.g. means) or other basic estimates (e.g. regression coefficient) AND variation (e.g. standard deviation) or associated estimates of uncertainty (e.g. confidence intervals)
☒	☐	For null hypothesis testing, the test statistic (e.g. *F*, *t*, *r*) with confidence intervals, effect sizes, degrees of freedom and *P* value noted<br>*Give P values as exact values whenever suitable.*
☒	☐	For Bayesian analysis, information on the choice of priors and Markov chain Monte Carlo settings
☒	☐	For hierarchical and complex designs, identification of the appropriate level for tests and full reporting of outcomes
☒	☐	Estimates of effect sizes (e.g. Cohen's *d*, Pearson's *r*), indicating how they were calculated

Our web collection on statistics for biologists contains articles on many of the points above.

Software and code

Policy information about availability of computer code

Data collection	FTIR: OPUS 5.5 (Bruker)<br><br>NMR: TopSpin 3.5 (Bruker Biospin)<br><br>CD: Chirascan 4.7 (Applied Photophysis)<br><br>AFM: Nanoscope 9.1 (Bruker)<br><br>Fluorescence microscopy: NIS elements BR software and Leica Application Suit X<br><br>SEM: SmartSEM
Data analysis	FTIR: OPUS 5.5 (Bruker), Kinetics (E. Goormaghtigh, E. Université Libre de Bruxelles, Belgium), Origin Pro 2018 95E (OriginLab Corporation).<br><br>WAXS: FIT2D 12.077 (Hammersley, ESRF Internal Report, ESRF98HA01T, FIT2D V9.129 Reference Manual V3.1, 1998)<br><br>NMR: TopSpin 3.5 (Bruker Biospin), CCPNMR 3.2 and 3.4 (Vranken, W.F. et al. Proteins-Structure Function and Bioinformatics 59, 687-696, 2005).

CD: Chirascan 4.7 (Applied Photophysis), BeStSel (Micsonai, A. et al Nucleic Acids Res 50, W90-W98, 2022).

AFM: Nanoscope Analysis 9.1 (Bruker)

Modelling/computational analysis: Consurf (Ashkenazy, H., et al. Nucleic Acids Research 44, W344-W350, 2016, https://consurf.tau.ac.il/), SWISS-MODEL (Waterhouse, A. et al. Nucleic Acids Research 46, W296-W303, 2018, https://swissmodel.expasy.org/), ZipperDB (Goldschmidt, L. et al. Proc. Natl. Acad. Sci. U.S.A. 107, 3487-3492, 2010, https://services.mbi.ucla.edu/zipperdb/), TANGO (Fernandez-Escamilla, A.M. et al. Nat Biotechnol 22, 1302-6, 2004, http://tango.crg.es/), Kyte & Doolittle hydropathicity (https://web.expasy.org/protscale/)

For manuscripts utilizing custom algorithms or software that are central to the research but not yet described in published literature, software must be made available to editors and reviewers. We strongly encourage code deposition in a community repository (e.g. GitHub). See the Nature Portfolio guidelines for submitting code & software for further information.

Data

Policy information about availability of data

All manuscripts must include a data availability statement. This statement should provide the following information, where applicable:

- Accession codes, unique identifiers, or web links for publicly available datasets
- A description of any restrictions on data availability
- For clinical datasets or third party data, please ensure that the statement adheres to our policy

Data will be will be made available through Zenodo. DOI: 10.5281/zenodo.11110183

Research involving human participants, their data, or biological material

Policy information about studies with human participants or human data. See also policy information about sex, gender (identity/presentation), and sexual orientation and race, ethnicity and racism.

Reporting on sex and gender	Not relevant
Reporting on race, ethnicity, or other socially relevant groupings	Not relevant
Population characteristics	Not relevant
Recruitment	Not relevant
Ethics oversight	Not relevant

Note that full information on the approval of the study protocol must also be provided in the manuscript.

Field-specific reporting

Please select the one below that is the best fit for your research. If you are not sure, read the appropriate sections before making your selection.

☒ Life sciences ☐ Behavioural & social sciences ☐ Ecological, evolutionary & environmental sciences

For a reference copy of the document with all sections, see nature.com/documents/nr-reporting-summary-flat.pdf

Life sciences study design

All studies must disclose on these points even when the disclosure is negative.

Sample size	At least two samples per technique
Data exclusions	No data was excluded
Replication	Each method was repeated at least twice. All attempts to repeat the experiments were successful.
Randomization	not relevant
Blinding	not relevant

Reporting for specific materials, systems and methods

We require information from authors about some types of materials, experimental systems and methods used in many studies. Here, indicate whether each material, system or method listed is relevant to your study. If you are not sure if a list item applies to your research, read the appropriate section before selecting a response.

Materials & experimental systems

n/a	Involved in the study
☒	☐ Antibodies
☒	☐ Eukaryotic cell lines
☒	☐ Palaeontology and archaeology
☒	☐ Animals and other organisms
☒	☐ Clinical data
☒	☐ Dual use research of concern
☒	☐ Plants

Methods

n/a	Involved in the study
☒	☐ ChIP-seq
☒	☐ Flow cytometry
☒	☐ MRI-based neuroimaging

Plants

Seed stocks	Not relevant
Novel plant genotypes	Not relevant
Authentication	Not relevant
